# Concomitant Salmonella and Leptospira Meningitis: A Rare Case Report

**DOI:** 10.7759/cureus.54611

**Published:** 2024-02-21

**Authors:** Rizwan Ullah, Aftab Ahmad, Yoalkris E Salcedo, Amir Hassan, Anuva Khanal, Aayush Chaulagain

**Affiliations:** 1 Internal Medicine, Hayatabad Medical Complex Peshawar, Peshawar, PAK; 2 Geriatrics, Cork University Hospital, Cork, IRL; 3 Surgery, Universidad Iberoamericana, Santo Domingo, DOM; 4 Emergency Medicine, Bronglais General Hospital, Aberystwyth, GBR; 5 Internal Medicine, Shaheed Ziaur Rahman Medical College and Hospital, Bogra, BGD

**Keywords:** bacterial meningitis, co-infection, meningitis, rare case report, neuroleptospirosis, salmonella and leptospira meningitis, concomitant

## Abstract

This study presents a unique case of concurrent salmonella and Leptospira meningitis in a 20-year-old woman with no prior medical history. Coinfection with endemic pathogens is plausible, especially in regions like Pakistan. While Salmonella meningitis is uncommon, it presents a significant medical emergency, particularly in immunocompromised adults. Neuroleptospirosis, though rare, can manifest in certain cases. The patient displayed persistent high fever, confusion, irritability, and a single seizure episode. Initial tests, including blood and cerebrospinal fluid (CSF) cultures and serological examinations, detected Salmonella typhi and positive leptospiral antibodies, respectively. Leptomeningeal enhancement was confirmed by an MRI. Treatment with azithromycin, meropenem, and ceftriaxone led to improvement after seven days. She was advised to complete a 28-day course for Salmonella meningitis. This case emphasizes the importance of considering multiple infectious causes, especially in endemic regions. Timely and thorough diagnostic evaluation, followed by appropriate antimicrobial therapy, is essential for effective management. Further research is warranted to enhance understanding of the epidemiology, clinical features, and optimal treatment strategies for such dual infections.

## Introduction

In developing nations, infectious diseases are the primary cause of illness and death. Among the prevalent infectious diseases in Pakistan are malaria, typhoid fever, leptospirosis, and dengue fever. Dealing with coinfections presents significant hurdles in both diagnosis and treatment, often leading to more adverse outcomes for patients compared to single infections [1, 2].

Leptospirosis, a zoonotic illness caused by pathogenic bacteria of the genus Leptospira, is primarily transmitted through direct or indirect contact with the urine of infected animals. Its clinical spectrum varies from mild anicteric leptospirosis, characterized by symptoms such as fever, headache, myalgia, sore throat, and cough, to more severe manifestations like Weil's syndrome, which includes jaundice, renal dysfunction, and bleeding tendencies, including pulmonary hemorrhage. Studies suggest that leptospirosis may contribute to 5-13% of all instances of aseptic meningitis [3,4]. Neurological involvement is reported in 10-15% of leptospirosis cases, with aseptic meningitis being the most common presentation. Although meningitis is recognized as a common feature of leptospirosis, it seldom presents as a primary neurological syndrome [5].

Salmonella infection is widely recognised as a leading cause of foodborne diarrhoea across the globe [6]. Salmonella meningitis although rare, however, when it does occur, it often poses a significant medical emergency, sometimes necessitating neurosurgical intervention, and can lead to considerable morbidity and mortality despite appropriate treatment [7]. The estimated prevalence of Salmonella meningitis is 4-6 cases per 100,000 individuals annually, particularly among those with normal immune function. This condition primarily affects children under the age of five, especially infants, whereas in adults, Salmonella meningitis is predominantly observed in individuals with compromised immune systems. The frequency of Salmonella meningitis among immunocompetent adults in South Asia remains uncertain, with limited information available in medical literature, primarily through isolated case reports [8,9].

Coinfection with endemic organisms is plausible in regions like Pakistan, where clinical symptoms and laboratory findings of various infectious diseases may overlap. When faced with multiple symptoms, physicians should consider Hickam's dictum (which suggests multiple reasons for symptoms) rather than Occam's razor (which favours a single cause). Here, we present the case of a 20-year-old woman with high fever, confusion, and irritability, diagnosed with concurrent salmonella and leptospiral meningitis. This is the first reported case of such a coinfection in medical literature.

## Case presentation

A 20-year-old woman with no prior medical issues presented with a persistent high fever of 104F for the past two weeks. She also experienced a single episode of seizures, along with confusion, irritability, and headaches over the last two days. Her family denied any recent travel, exposure to sick individuals, or gastrointestinal symptoms. Physical examination revealed a feverish appearance with pallor, a malar rash, and dry mucous membranes, but normal sclera. Neurological examination showed disorientation in time place and person, normal pupillary size and reaction. No focal abnormalities. No signs of meningitis (neck stiffness, Kernig's sign). Abdominal examination revealed soft and tender hepatomegaly, while lung examination indicated clear chest sounds with normal breathing patterns.

We conducted a comprehensive set of investigations based on the differential diagnosis, which included tests for renal and liver function, coagulation profiles that were normal and malarial parasites, dengue serology, Brucella serology, and antinuclear antibodies (ANA), all of which returned negative. Further tests were initiated, including leptospiral serology, blood culture and sensitivity, routine examination and culture of cerebrospinal fluid (CSF), and an ultrasound of the abdomen and pelvis. Additionally, an MRI of the brain with contrast was scheduled. Some laboratory investigations are summarized in Table 1.

**Table 1:** Investigations performed during hospital stay CRP: C-reactive protein; ESR: Erythrocyte sedimentation rate; CSF: Cerebrospinal fluid; LDH: Lactate dehydrogenase

Investigations	Reference range	Results
White cell count (x10^3^/mcL)	4-11	6.31
Hemoglobin (g/dL)	11.5-17.5	8.22
Platelet count (x10^3^/mcL)	150-450	138
Mean Corpuscular volume (fL)	76-96	82.4
Hematocrit (%)	36-54	24.7
CRP (mg/dL)	<0.5	1.88
ESR (mm/hour)	0-20	17
Ferritin (ng/mL)	13-150	296
Anti-leptospiral IgM (Serum and CSF)	<0.9	1.89
LDH (U/L)	80-235	404

**Figure 1:**
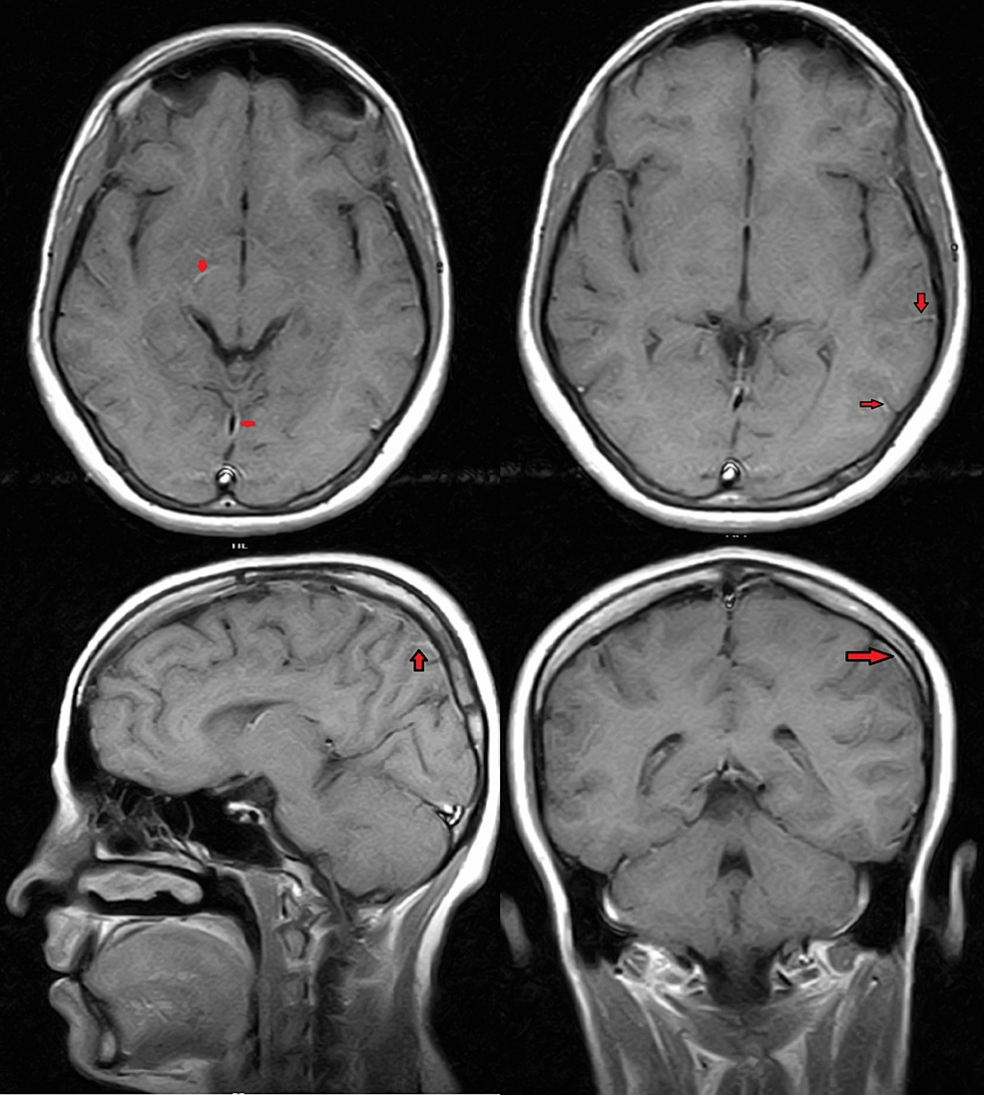
Post-contrast T1-weighted images show mild leptomeningeal enhancement (shown by red arrows).

After initiating empirical therapy (Injection ceftriaxone 2 gm/day in two equally divided doses, Inj. vancomycin 1.5 gm/day in three equally divided doses) for bacterial meningoencephalitis, there was no improvement observed after five days. The ultrasound of the abdomen revealed hepatomegaly, with a diameter of 15 cm at the midclavicular line. Leptospira serology and CSF returned positive for IgM by immunosorbent assay (ELISA), and the CSF routine examination indicated straw-coloured fluid with 550 white blood cells (WBC) per cubic millimetre and 10 red blood cells (RBC) per cubic millimetre, with protein levels at 330 mg/dL and glucose at 6 mg/dL, predominant neutrophilia. Both blood and CSF cultures revealed Salmonella typhi, which was found to be sensitive to azithromycin, meropenem. Additionally, MRI brain post-contrast T1-weighted images displayed mild leptomeningeal enhancement, as illustrated in Figure 1.

Based on cerebrospinal fluid findings, positive serology for Leptospira, MRI results, and cultures from blood and CSF, we diagnosed this as a case of concurrent salmonella and leptospiral meningitis.

The patient was initiated on a treatment regimen consisting of azithromycin and meropenem for Salmonella meningitis, supplemented with ceftriaxone for coverage against leptospira, considering positive CSF and serology for leptospira. After seven days of this treatment, the patient showed improvement in the resolution of her fever. The patient was discharged on home medications (Tab. azithromycin 500 mg once daily) to complete Salmonella meningitis course of 28 days. She was advised to follow up to monitor for any potential complications or sequelae of the disease.

## Discussion

Typhoid fever and leptospirosis are prevalent infections in Pakistan, and their concurrent occurrence poses significant challenges in diagnosis and treatment, potentially resulting in severe outcomes including disability and death. Reports of coinfection with both S. typhi and Leptospira have been documented globally, including in countries such as the USA, Denmark, Egypt, Tanzania, India, and Korea. In the USA, a 16-year-old boy was diagnosed with Salmonella osteomyelitis along with leptospirosis. The diagnosis was confirmed by culturing S. typhi from an abscess in the intervertebral disc space. The Salmonella strain was found to be susceptible to penicillin and quinolones [10]. Rönsholt et al. documented a case of coinfection with Salmonella typhi and Leptospira in a 56-year-old man who was hospitalized with septicemia following occupational exposure. Salmonella typhi was isolated from stool culture, while Leptospira was identified using polymerase chain reaction (PCR) [11]. Parker et al. conducted a study in Egypt, where they identified just a single confirmed case of coinfection with both Salmonella typhi and Leptospira among 1510 patients presenting with acute febrile illness, based on positive cultures for both organisms [12]. Biggs et al. conducted research in Tanzania and found that among 40 confirmed cases of leptospirosis, three individuals showed indications of coinfection with Salmonella typhi [13]. Sahu et al. in India documented a case where Salmonella typhi induced a splenic abscess in a patient concurrently suffering from leptospirosis. Notably, the Salmonella typhi strain was susceptible to treatment with ceftriaxone [14]. Song et al. in Korea documented a case involving a 37-year-old man whose blood culture revealed Salmonella typhi infection alongside positive serological findings for Leptospira [15].

We presented a 20-year-old woman who exhibited symptoms such as high-grade fever, confusion, irritability, and a single seizure episode. Subsequent laboratory investigations revealed positive serology for Leptospira, along with positive blood and CSF cultures for Salmonella typhi. Additionally, MRI brain scans indicated mild leptomeningeal enhancement. This led to the diagnosis of concomitant salmonella and Leptospira meningitis in the patient.

Neurological symptoms in leptospirosis varied, but prevalent presentations included changes in consciousness, a profoundly comatose state, and sudden symptomatic seizures, as observed in a study involving 31 patients diagnosed with leptospirosis. Neurological symptoms are observed in approximately 10-15% of individuals with leptospirosis, while indications of meningeal irritation are present in 80% of neuroleptospirosis case. Our patient did not exhibit clinical signs of meningitis. Initial presentation with primary neuroleptospirosis is rare. Without hepatic or renal involvement, diagnosing leptospirosis can be delayed. Aseptic meningitis is the most frequent manifestation of neuroleptospirosis, representing 5-13% of all cases of aseptic meningitis in one study [3,4].

Salmonella meningitis is uncommon, with an estimated incidence of only 4-6 cases per 100,000 individuals each year, especially among those who have a healthy immune system. Nevertheless, when it does happen, it frequently presents a serious medical crisis, occasionally requiring neurosurgical procedures, and can result in substantial illness and death despite receiving suitable medical care. This disease primarily impacts children below the age of five, particularly infants, whereas in adults, Salmonella meningitis is mainly observed in individuals with weakened immune systems [7-9]. The prevalence of Salmonella meningitis in immunocompetent adults in South Asia is not well established, as there is limited information in medical literature, mostly derived from isolated case reports.

Leptospira can be detected in cerebrospinal fluid (CSF), showing normal cytology and biochemistry, except for increased CSF pressure during the leptospiremic phase. In the immune phase, elevated CSF pressure, lymphocytic pleocytosis, elevated protein levels, and normal glucose levels may be observed. Additionally, anti-Leptospira antibodies may be present in the CSF during this stage. Molecular diagnostic techniques have demonstrated effectiveness in the early detection of leptospiral DNA in CSF. CSF analysis typically reveals a predominance of lymphocytes in later stages, although initially, polymorphonuclear leukocytes may be more common. Blood serology by immunosorbent assay (ELISA) is diagnostic for leptospirosis. Salmonella can be diagnosed with a blood culture. Additional investigations include a CSF study and brain imaging [3,4,8]. In our case, we conducted tests on cerebrospinal fluid (CSF) and serum for leptospiral antibodies, and both yielded positive results. Additionally, blood culture revealed the presence of Salmonella typhi. MRI brain scans exhibited enhancement of the meninges.

The treatment options for neuroleptospirosis have not been extensively researched, but penicillin, cephalosporins, doxycycline, and chloramphenicol are commonly recommended for managing leptospirosis. In northern Pakistan, cases of extensively drug-resistant (XDR) S. typhi have been documented, exhibiting susceptibility solely to azithromycin and meropenem [4,16].

Our case was exceptionally rare, as there have been no similar cases documented in medical literature till date. We initiated treatment for our patient with azithromycin, meropenem, and ceftriaxone, and she exhibited a positive response.

## Conclusions

In conclusion, we presented a unique case of a 20-year-old woman with concurrent salmonella and Leptospira meningitis, a rare occurrence with no previous documented cases in the medical literature. This case highlights the importance of considering multiple infectious etiologies, especially in regions where such infections are endemic. Prompt and comprehensive diagnostic evaluation, including serological and microbiological testing, along with imaging studies, played a crucial role in arriving at an accurate diagnosis. Effective management of such cases requires a multidisciplinary approach and the early initiation of appropriate antimicrobial therapy based on susceptibility testing. Further research is warranted to better understand the epidemiology, clinical manifestations, and optimal treatment strategies for dual infections such as this, particularly in regions with high burdens of both salmonella and Leptospira infections.
